# Supplementary material for: Contextual cueing of visual search reflects the acquisition of an optimal, one-for-all oculomotor scanning strategy
Source: Commun Psychol. 2023 Sep 20;1:20. doi: 10.1038/s44271-023-00019-8 (PMC11332235; doi:10.1038/s44271-023-00019-8)
Supplement: Supplementary file 1 — Supplementary Information [file 44271_2023_19_MOESM1_ESM.pdf]

Supplementary Information for:

"Contextual cueing of visual search reflects the acquisition of an optimal, one-for-all oculomotor scanning strategy" by:

Seitz, W., Zinchenko, A., Müller, H.J., and Geyer, T.

Supplementary Figure 1

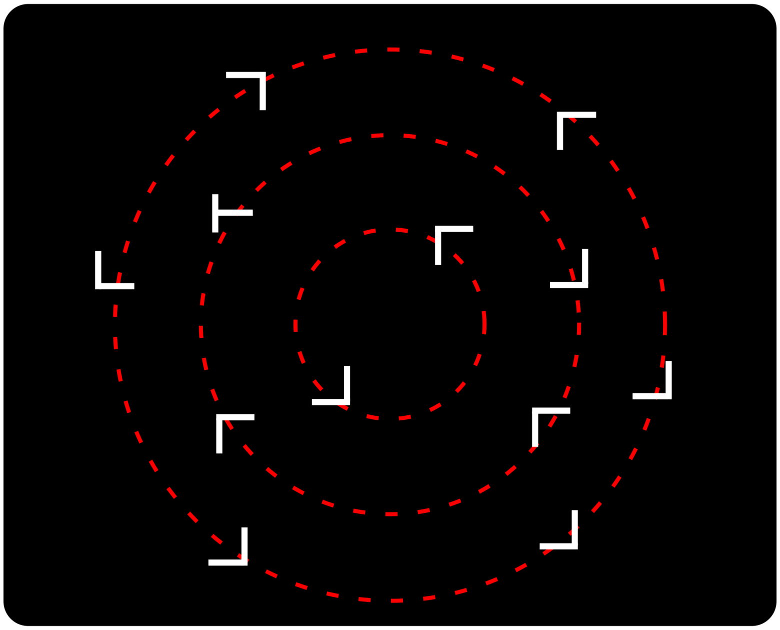

Supplementary Figure 1. Example search display presenting a repeated target-distractor configuration. Note that the red dashed circles, depicting the three concentric rings around which the search items were arranged, were not shown in the actual search displays. Targets in repeated and non-repeated displays were always presented on the second (middle) ring at one of a total of N=8 different positions distributed equally across the four display quadrants; i.e., each display quadrant contained one repeated and one (different) non-repeated target position.

Supplementary Figure 2

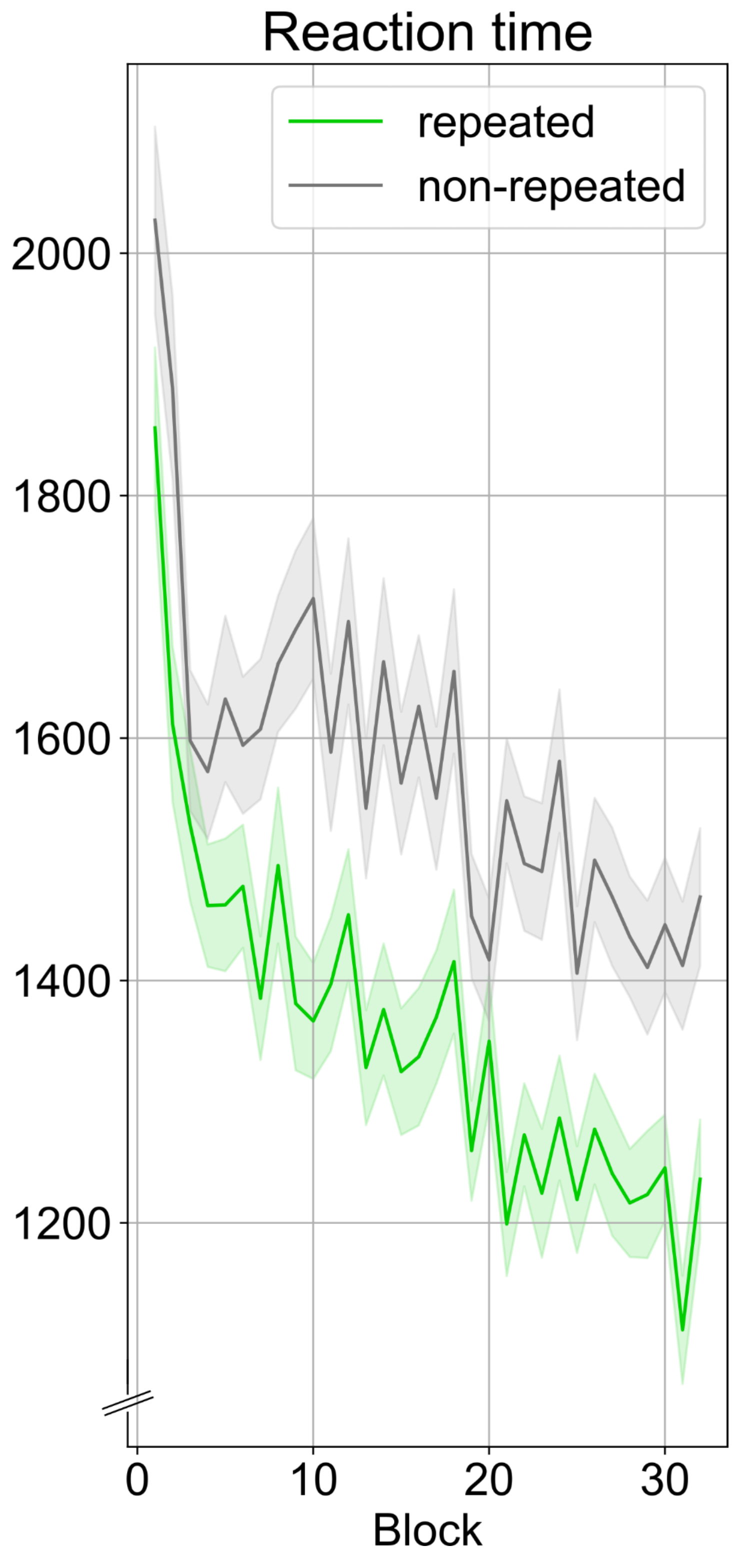

Supplementary Figure 2. Average reaction times (in ms) as a function of time on task (block). The shaded areas represent the standard error of the mean.

Supplementary Figure 3

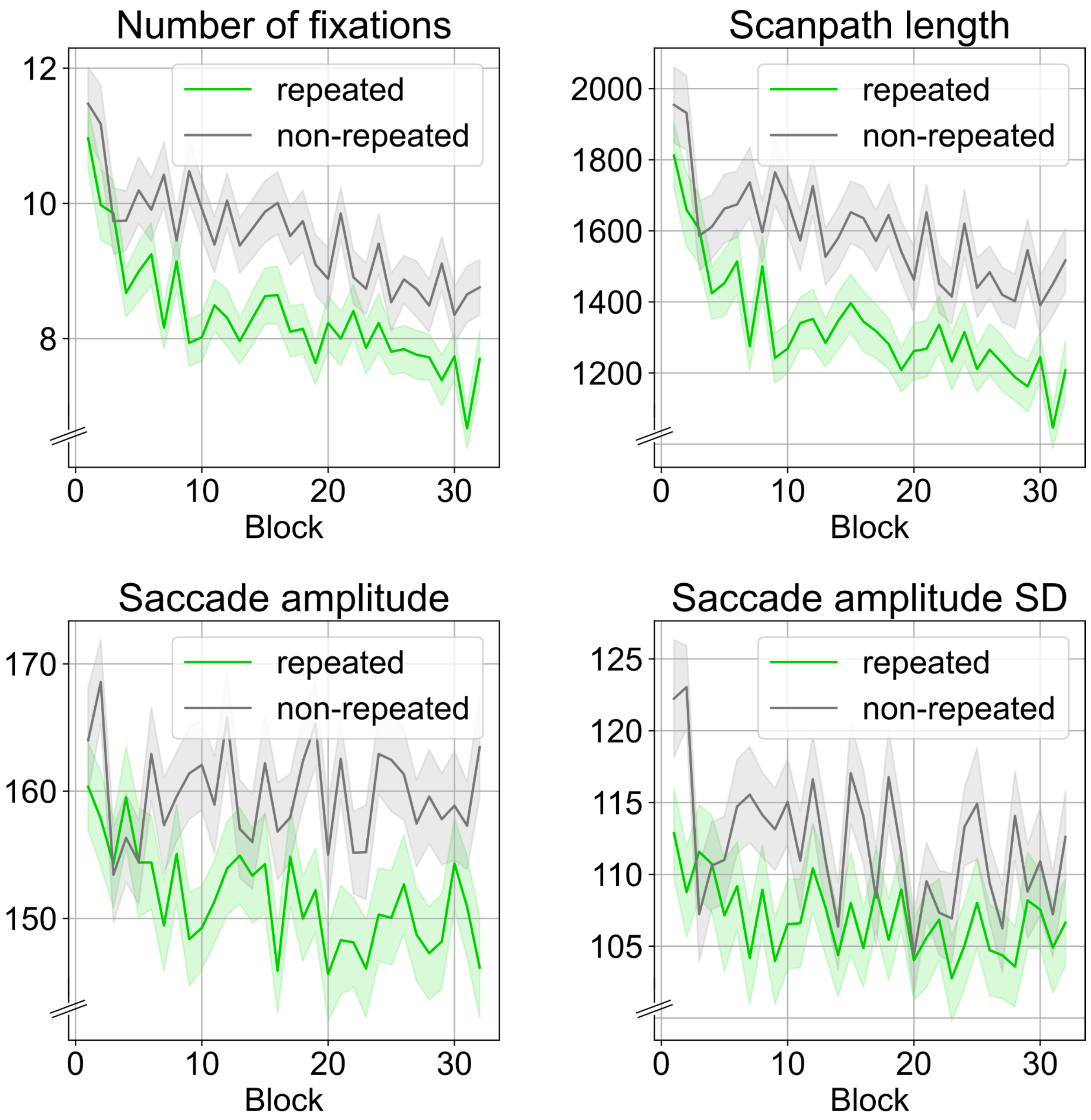

Supplementary Figure 3. Four oculomotor measures as a function of block, namely: the number of fixations, the scanpaths' absolute length (in pixel), the saccade amplitude (in pixel) as well, as the saccade amplitude's standard deviation (in pixel). The shaded areas represent the standard error of the mean.

Supplementary Figure 4

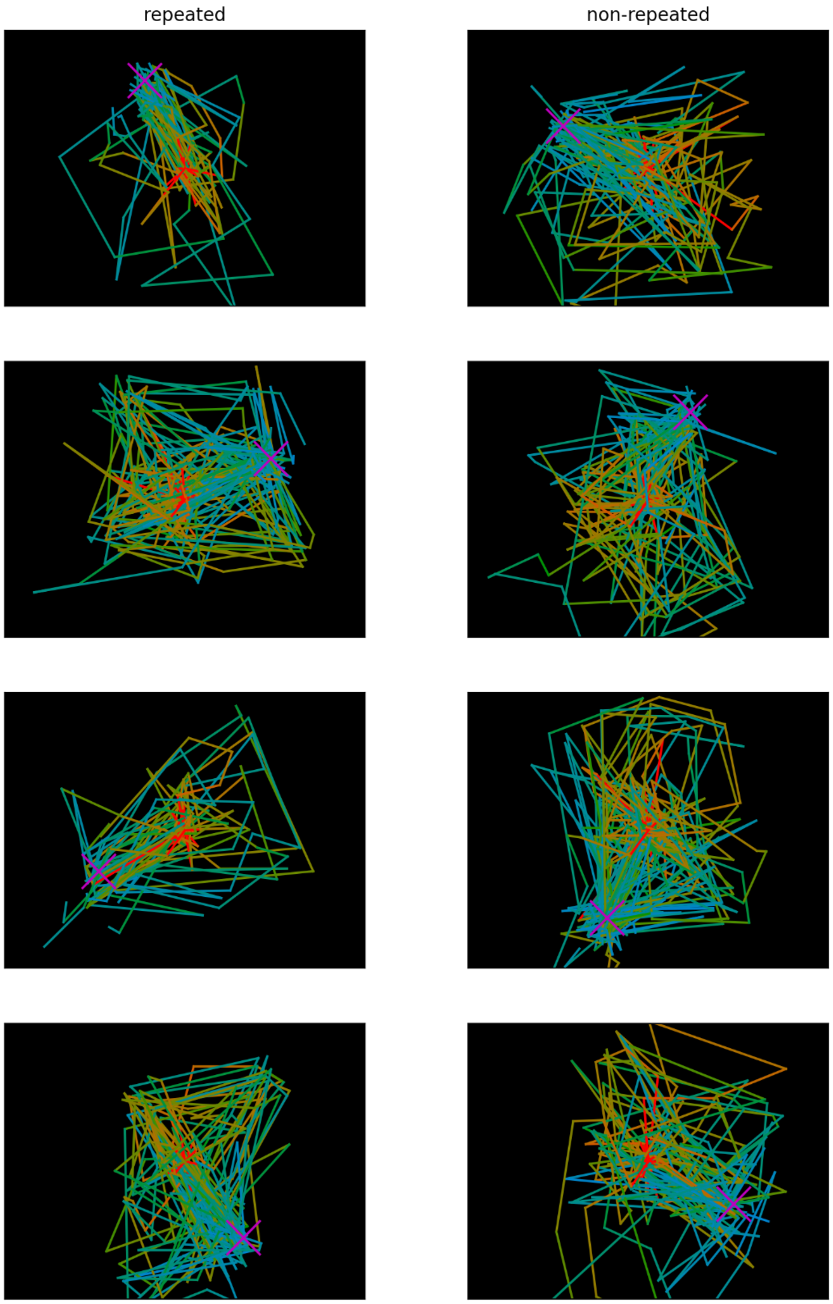

Supplementary Figure 4. Depiction of oculomotor scanpaths (of N=46 participants) for the four repeated and the four non-repeated displays (left and right columns, respectively, with the rows showing each of the four target positions in these displays) in the last block of the search task (i.e., after the maximal time of learning). Scanpaths gradually change color from red to blue, marking the first and last saccade in a trial, respectively; the target location is indicated by a purple cross. The lines representing individual participants' scanpaths are more similar for repeated as compared to non-repeated displays: Specifically, there is a higher proportion of parallel saccade vectors in repeated displays (indicative of similar decisions as to where to deploy attention on a fixation-to-fixation basis) and a stronger 'clustering' of colors (indicative of a higher similarity of the spatio-temporal structure of the overall sequence of fixations).

Supplementary Figure 5

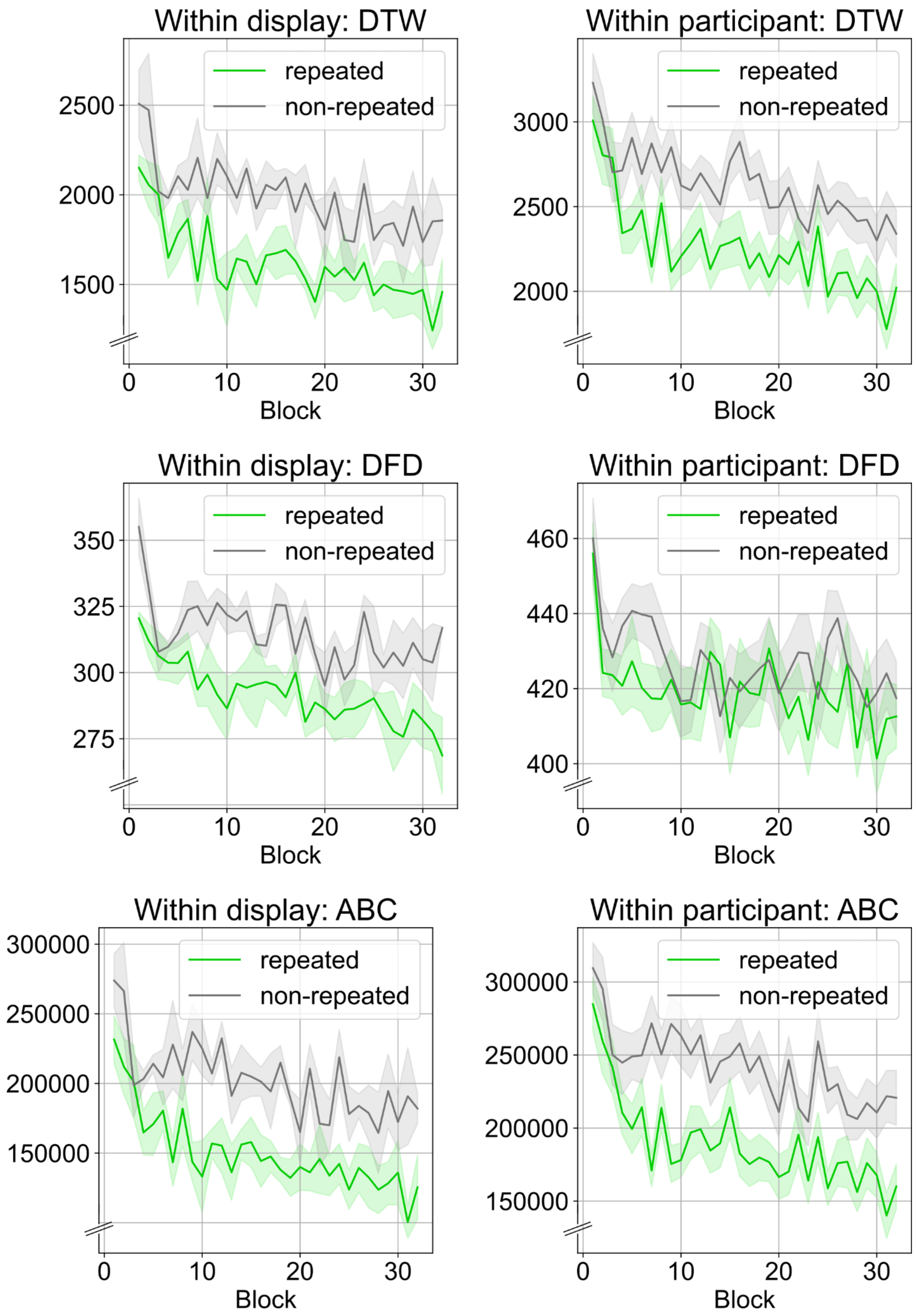

Supplementary Figure 5. Similarity between scanpaths within displays and within participants for three measures: Dynamic Time Warping (in pixel), Discrete Fréchet Distance (in pixel) and Area Between Curves (in square-pixel) as a function of block. The shaded areas represent the standard error of the mean.

Supplementary Methods

We used mixed effects models in which we included participants and/or display as random effects (for the reasoning, see Methods). Here, we outline a detailed model structure for each analysis.

Oculomotor measures:
For the oculomotor measures (reaction time, number of fixations, mean saccade amplitude and standard deviation of saccade amplitude), the fixed effects were block and context. As random effects were included the intercepts of participants and quadrants in order to account for the fact that each participant saw the same set of displays. The R-code used was the following:

```
model <- lmer(DV ~ context * block + (1|participant)+(1|quadrant), data = df),
```

with DV being our oculomotor measures.

Within participant similarity:
For the analyses within participant, we included block and context as fixed effects and the participants' intercepts as random effects. The R-code used was:

```
model <- lmer(similarity ~ context * block + (1|participant), data = df),
```

with similarity being one of our three similarity measures.

Within display similarity:
For the analyses within participant, we included block and context as fixed effects and the intercepts of the target quadrants as random effects. The R-code used was as follows:

```
model <- lmer(similarity ~ context * block + (1|quadrant), data = df),
```

with similarity being one of our three similarity measures.

No model had convergence issues. Therefore, no simplifying assumptions were necessary.

Supplementary Table

The table summarizes the schemes for computing similarity measures for sets of either repeated or non-repeated contexts. Pairs of scanpaths were compared at different levels relating to 1) similarity of fixational sequences originating from an individual display when viewed by pairs of different participants ("Within display across participant"); 2) similarity of scanpaths for an individual participant when viewing (pairs of) different displays ("Across display, within participant"). What is presented are the scanpath comparisons per block for three hypothetical participants as well as three hypothetical repeated and three non-repeated displays. Numbers denote individual participants' identities and, respectively, the target quadrants in individual displays; thus, e.g., "Re_1 Pa_1" corresponds to a repeated display (abbreviated Re) with the same target quadrant as in non-repeated display (No) "No_1 Pa_1" for the same participant (Pa) Pa1. –

A)

**Within display across participant**	**Repeated 1**	**Repeated 2**	**Repeated 3**	**Non-Repeated 1**	**Non-Repeated 2**	**Non-Repeated 3**
**Participant 1**	Re_1 Pa_2, Re_1 Pa_3	Re_2 Pa_2, Re_2 Pa_3	Re_3 Pa_2, Re_3 Pa_3	No_1 Pa_2, No_1 Pa_3	No_2 Pa_2, No_2 Pa_3	No_3 Pa_2, No_3 Pa_3
**Participant 2**	Re_1 Pa_1, Re_1 Pa_3	Re_2 Pa_1, Re_2 Pa_3	Re_3 Pa_1, Re_3 Pa_3	No_1 Pa_1, No_1 Pa_3	No_2 Pa_1, No_2 Pa_3	No_3 Pa_1, No_3 Pa_3
**Participant 3**	Re_1 Pa_1, Re_1 Pa_2	Re_2 Pa_1, Re_2 Pa_2	Re_3 Pa_1, Re_3 Pa_2	No_1 Pa_1, No_1 Pa_2	No_2 Pa_1, No_2 Pa_2	No_3 Pa_1, No_3 Pa_2

**Across display, within participant**	**Repeated 1**	**Repeated 2**	**Repeated 3**	**Non-Repeated 1**	**Non-Repeated 2**	**Non-Repeated 3**
**Participant 1**	Pa_1 Re_2, Pa_1 Re_3	Pa_1 Re_1, Pa_1 Re_3	Pa_1 Re_1, Pa_1 Re_2	Pa_1 No_2, Pa_1 No_3	Pa_1 No_1, Pa_1 No_3	Pa_1 No_1, Pa_1 No_2
**Participant 2**	Pa_2 Re_2, Pa_2 Re_3	Pa_2 Re_1, Pa_2 Re_3	Pa_2 Re_1, Pa_2 Re_2	Pa_2 No_2, Pa_2 No_3	Pa_2 No_1, Pa_2 No_3	Pa_2 No_1, Pa_2 No_2
**Participant 3**	Pa_3 Re_2, Pa_3 Re_3	Pa_3 Re_1, Pa_3 Re_3	Pa_3 Re_1, Pa_3 Re_2	Pa_3 No_2, Pa_3 No_3	Pa_3 No_1, Pa_3 No_3	Pa_3 No_1, Pa_3 No_2
