# Supplementary material for: Contextual cueing of visual search reflects the acquisition of an optimal, one-for-all oculomotor scanning strategy
Source: Commun Psychol. 2023 Sep 20;1:20. doi: 10.1038/s44271-023-00019-8 (PMC11332235; doi:10.1038/s44271-023-00019-8)
Supplement: Supplementary file 3 — Reporting Summary [file 44271_2023_19_MOESM3_ESM.pdf]

nature portfolio

Corresponding author(s): Werner Seitz

Last updated by author(s): Aug 20, 2023

Reporting Summary

Nature Portfolio wishes to improve the reproducibility of the work that we publish. This form provides structure for consistency and transparency in reporting. For further information on Nature Portfolio policies, see our Editorial Policies and the Editorial Policy Checklist.

Statistics

For all statistical analyses, confirm that the following items are present in the figure legend, table legend, main text, or Methods section.

n/a	Confirmed	
☐	☒	The exact sample size (*n*) for each experimental group/condition, given as a discrete number and unit of measurement
☐	☒	A statement on whether measurements were taken from distinct samples or whether the same sample was measured repeatedly
☐	☒	The statistical test(s) used AND whether they are one- or two-sided<br>*Only common tests should be described solely by name; describe more complex techniques in the Methods section.*
☒	☐	A description of all covariates tested
☒	☐	A description of any assumptions or corrections, such as tests of normality and adjustment for multiple comparisons
☐	☒	A full description of the statistical parameters including central tendency (e.g. means) or other basic estimates (e.g. regression coefficient) AND variation (e.g. standard deviation) or associated estimates of uncertainty (e.g. confidence intervals)
☐	☒	For null hypothesis testing, the test statistic (e.g. *F*, *t*, *r*) with confidence intervals, effect sizes, degrees of freedom and *P* value noted<br>*Give P values as exact values whenever suitable.*
☐	☒	For Bayesian analysis, information on the choice of priors and Markov chain Monte Carlo settings
☒	☐	For hierarchical and complex designs, identification of the appropriate level for tests and full reporting of outcomes
☒	☐	Estimates of effect sizes (e.g. Cohen's *d*, Pearson's *r*), indicating how they were calculated

Our web collection on statistics for biologists contains articles on many of the points above.

Software and code

Policy information about availability of computer code

Data collection	Matlab 2012a, Psychtoolbox version 3.0.13
Data analysis	R3.4.3, Python 3.7.4

For manuscripts utilizing custom algorithms or software that are central to the research but not yet described in published literature, software must be made available to editors and reviewers. We strongly encourage code deposition in a community repository (e.g. GitHub). See the Nature Portfolio guidelines for submitting code & software for further information.

Data

Policy information about availability of data

All manuscripts must include a data availability statement. This statement should provide the following information, where applicable:

- Accession codes, unique identifiers, or web links for publicly available datasets
- A description of any restrictions on data availability
- For clinical datasets or third party data, please ensure that the statement adheres to our policy

The raw data that support the analysis and results are publicly accessible at https://osf.io/snjpk/

Human research participants

Policy information about studies involving human research participants and Sex and Gender in Research.

Reporting on sex and gender	Self-reported gender was collected, however, for this study, no further analyses based on sex and/or gender have been conducted. Since there are no studies explicitly investigating the influence of sex/gender on contextual cueing, we primarly collected this information to ensure that we would not obtain an unexpected, such as e.g. an all-male sample.
Population characteristics	We collected handedness and age, however no further analyses have been conducted in this study based on this information.
Recruitment	Participants were recruited through the mailing list of the department. We accepted everyone who came until we reached 50 participants, so there was no self-selection bias
Ethics oversight	Ethics approval of the full study protocol was obtained from the LMU's Department of Psychology on 11.05.2018 (approval number/ ID: GE 1889/4-2). Further, informed consent was obtained from each participant.

Note that full information on the approval of the study protocol must also be provided in the manuscript.

Field-specific reporting

Please select the one below that is the best fit for your research. If you are not sure, read the appropriate sections before making your selection.

☐ Life sciences ☒ Behavioural & social sciences ☐ Ecological, evolutionary & environmental sciences

For a reference copy of the document with all sections, see nature.com/documents/nr-reporting-summary-flat.pdf

Behavioural & social sciences study design

All studies must disclose on these points even when the disclosure is negative.

Study description	The current work is a quantitative study, using reaction time and (numerical) oculomotor parameters as dependent variables.
Research sample	All participants were LMU bachelor and master students, recruited by the mailing list. None had neurological/psychological disorders, all had normal or corrected to normal vision. Since contextual cueing is known to be independent of IQ (Merill et al., 2014), emerge early and be spared during ageing (Dixon et al., 2010; Howard et al., 2004), we have no reason to assume that the sample is not representative.
Sampling strategy	The sample size was determined based on previous contextual cueing studies using relatively large sample sizes (e.g., Vadillo, Malejka, Lee, Dienes & Shanks, 2021; Peterson, Mead, Kelly, Esser-Adomako & Blumberg, 2022) in order to obtain reasonably stable estimates of contextual cueing.
Data collection	Eye-tracking and a computer were used to obtain the dependent measures, for details, see Methods. Participants were seated in a dimly lit room and performed the experiment undisturbed. They were allowed to take breaks between the experimental blocks.
Timing	Data collection started on the 04.11.2020 and finished on 15.12.2020. There were no significant gaps.
Data exclusions	We did not exclude anyone. We collected data from 50 participants ow which 4 datasets were corrupted (no data were recorded in the eye tracker) and these were not included into the analysis.
Non-participation	None of the participants declined or dropped out.
Randomization	The study had a within-design, so no group allocation was implemented.

Reporting for specific materials, systems and methods

We require information from authors about some types of materials, experimental systems and methods used in many studies. Here, indicate whether each material, system or method listed is relevant to your study. If you are not sure if a list item applies to your research, read the appropriate section before selecting a response.

Materials & experimental systems

n/a	Involved in the study
☒	☐ Antibodies
☒	☐ Eukaryotic cell lines
☒	☐ Palaeontology and archaeology
☒	☐ Animals and other organisms
☒	☐ Clinical data
☒	☐ Dual use research of concern

Methods

n/a	Involved in the study
☒	☐ ChIP-seq
☒	☐ Flow cytometry
☒	☐ MRI-based neuroimaging
